# Age-related changes in the risk of high blood pressure

**DOI:** 10.3389/fcvm.2022.939103

**Published:** 2022-09-15

**Authors:** Weibin Cheng, Yumeng Du, Qingpeng Zhang, Xin Wang, Chaocheng He, Jingjun He, Fengshi Jing, Hao Ren, Mengzhuo Guo, Junzhang Tian, Zhongzhi Xu

**Affiliations:** ^1^Department of Health Management, Guangdong Second Provincial General Hospital, Guangzhou, China; ^2^Institute for Healthcare Artificial Intelligence Application, Guangdong Second Provincial General Hospital, Guangzhou, China; ^3^School of Data Science, City University of Hong Kong, Hong Kong, Hong Kong SAR, China; ^4^School of Artificial Intelligence, Tianjin University, Tianjin, China; ^5^School of Information Management, Wuhan University, Wuhan, China

**Keywords:** age-related trend, high blood pressure, generalized additive models, South China, heart failure

## Abstract

**Background and aims:**

Understanding the age-related trend of risk in high blood pressure (BP) is important for preventing heart failure and cardiovascular diseases. But such a trend is still underexplored. This study aims to (a) depict the relationship of BP patterns with age, and (b) understand the trend of high BP prevalence over time in different age groups.

**Materials and methods:**

Health check-up data with an observational period of 8 years (January 1, 2011, to December 31, 2018) was used as the data source. A total of 71,468 participants aged over 18 years old with complete information on weight, height, age, gender, glucose, triglyceride, total cholesterol, systolic (SBP), and diastolic blood pressure (DBP) were included for analysis. Generalized additive models were adopted to explore the relationship between the risk of high BP and age. Variance analysis was conducted by testing the trend of high BP prevalence in age groups over time.

**Results:**

Risk of high SBP showed a continuous rise from age 35 to 79 years and a concurrent early increase in the risk of high DBP; after age 50–65 years, high DBP risk declined. The risk of SBP rises linearly with age for men, whereas increases non-linearly for women. In addition, a significant increasing trend of high SBP risk among middle-aged people was found during the past decade, men experienced a later but longer period of increase in high SBP than women.

**Conclusion:**

The high SBP risk progresses more rapidly in the early lifetime in women, compared to the lifetime thereafter. Thresholds of increasing trend of SBP suggest a possible need for hypertension screening in China after the age of 40.

## Background

High blood pressure (BP), including high systolic blood pressure (SBP) and high diastolic blood pressure (DBP), has been recognized as triggers of the leading cause of mortality in the 21st century (1). It is estimated that an annual death of 7.7–10.4 million were associated with elevated blood pressure world-wide (2). High BP is also associated with the strongest evidence for causation of cardiovascular diseases (CVD) (3). The most important one was based on experience in 61 cohort studies that provided 12.7 million person-years of observation. The risk of CVD increased steadily with progressively higher levels of baseline SBP and DBP (4). For example, at ages 40–69 years, each difference of 20 mm Hg usual SBP is associated with more than a twofold difference in the stroke death rate (4).

Blood pressure is highly age-dependent. Existing literature has documented the age-related trend of absolute BP, which shows a linear rise of SBP with age after 30–40 years old (5–8) and reaching a plateau in late life (9). For DBP, an inverse U-shaped age-related trend was identified, with its peak at 40–60 years old (5–8). However, literature on this line manually split continuous age into discrete age groups, bringing in artificial influence. Moreover, age-BP trend is formulated by counting the number of subjects that fall into handcrafted age intervals. Therefore, there is a lack of adjustment for confounding factors. We found only one recent study (10) that enables an arbitrary complex age-BP relationship when investigating the life course BP trajectory, by using restricted cubic splines-based regression models. The authors focused on exploring sex differences in blood pressure trajectories over a lifetime, but the investigation of the protective or risk effect of aging and other covariates on high BP risk is lacking.

Previous literature also reported mixed findings about the high BP trend in developing countries (11–13): Studies conducted in Mexico and China reported that hypertension is shifting toward younger ages over time (11), whereas researchers from Iran found that younger generations are at lower risk of developing hypertension (13).

Against this backdrop, we present an analysis of 8 years’ routine health check-up data with the aim of (a) articulating the relationship of BP patterns with age using advanced modeling techniques, and (b) understanding the trend of blood pressure over time in different age groups. For task (a), we employed generalized additive models (GAM) with interaction items (GA^2^M). Compared to linear models and mixed linear models which put strict constraints on the potentially complex relationships between a dependent variable and independent variables, GA^2^M permits arbitrary complex relationships between the two. Thus, it is potentially capable of sophisticatedly depicting the age-related BP elevation risk. For task (b), the large-scale dataset used in this study creates a unique opportunity to further the current understanding of the trend of high BP prevalence over time in the general population in south China. The findings of this article could provide insights that can aid high BP prevention.

## Participants and methods

### Data source

**FIGURE 1:**
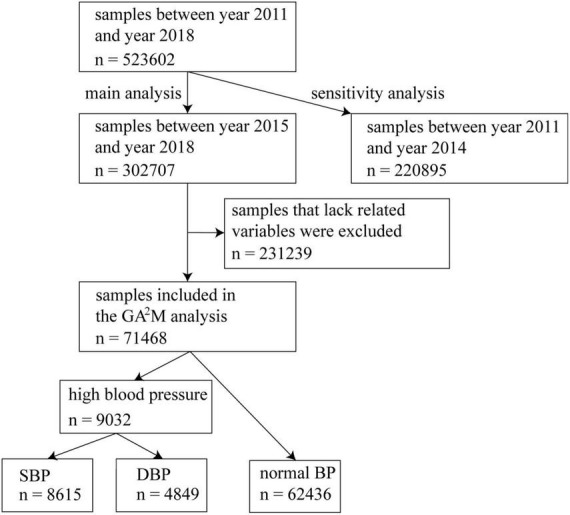
Sample selection process.

This study followed a cross-sectional design. Health check-up data with an 8-year (January 1, 2011, to December 31, 2018) observational period from Guangdong Second Provincial General Hospital, Guangdong, China was used in this study. Records from January 1, 2015, to December 31, 2018 are used in the main analysis. Records in the earlier period (January 1, 2011, to December 31, 2014) were used for model validation. A health check-up was conducted in the physical examination department in Guangdong Second Provincial General Hospital. Check-up records would be eligible for analysis if the participants were aged 18 years or older, and had complete information on the participants’ weight, height, age, gender, glucose (GLU), triglyceride (TG), total cholesterol (TC), and systolic blood pressure and diastolic blood pressure. Figure 1 demonstrates the sample selection process.

### Variables and measurements

#### Outcome variable

The outcome was defined as a binary variable (i.e., whether the participants have high SBP/DBP) according to their BP measurements. Participants were told not to drink a caffeinated beverage or smoke during the 30 min before the BP examination. They were also required to sit quietly for 5 min before the test begins. To measure BP, participants shall sit in a chair with feet on the floor and right arm supported such that the elbow is at about heart level. A second measurement would be taken if the first reading is above the normal limit (i.e., >90 mmHg for DBP or >140 mmHg for SBP). An automatic electronic blood pressure monitor was applied to BP test. Only one reading for SBP/DBP measurement was recorded for each person, the second reading would be put down if the blood pressure was measured twice. Participants with high SBP/DBP were annotated as positive cases, with a dependent variable (*y*1), those with SBP/DBP below the normal limit are classified as negative counterparts (*y*0).

#### Independent variable

The age of the participants being recorded while taking the physical examination was regarded as the main independent variable. Other independent covariates included body mass index (i.e., person’s weight in kilograms divided by the square of height in meters), glucose (GLU), triglyceride (TG), and total cholesterol (TC). All the independent variables were used as continuous variables.

### Analysis

#### Empirical analysis

We conducted the empirical analysis by calculating the high SBP/DPB prevalence (without any adjustment for confounding influences), which is simply dividing the number of high SBP/DBP individuals by the total research samples for each age group. We also summarized the characteristics of the research samples. Age, BMI, GLU, and TG are continuous variables, for which we reported the mean and standard deviation (SD). Gender is a binary feature, so we calculated and reported the number and percentage of men. Mann–Whitney *U* test and χ^2^ test with Yates’ correction were performed to explore the statistical significance of the continuous variables and the binary variable, respectively.

#### Main analysis

We formulated the first task of this study, which aims to explore the relationship between BP elevation risk and age, as a binary classification problem. The relationship was adjusted for covariates including Body Mass Index (BMI), glucose (GLU), triglyceride (TG), and total cholesterol (TC), to control the confounders. A generalized additive model (GAM) was adopted to explore the relationship between dependent and independent variables. A standard GAM has the form:


$$g\,\left(y\right)=\beta_{0}+\sum f_{i}\,\left(x_{i}\right)$$

Where *g*(⋅) is the link function and *f*_*i*_(⋅) is the boosted trees-based shape function for term *i*. Compared to the standard logistic regression model, which has a form of *g*(*y*)1/1 + *e*^−*w*_*i*_*x*_*i*_^, GAM permits arbitrary complex relationships between individual features and the target to be captured while avoiding overfitting. Therefore, it is a powerful tool to help us understand the age-related BP elevation risk. Pairwise interactions can be added to standard GAMs, leading to a model called GA^2^M. We also performed the standard logistic regression analysis using the same samples and covariables of the GA^2^M analysis. We adopted the Receiver Operating Characteristic of the Area Under the Curve (ROCAUC, also known as c-statistic) to evaluate the performances of GA^2^M and the logistic regression model.

To cope with the second task that focuses on understanding the overall trend of high BP prevalence over the 8-year observational period within the different age groups, variance analysis was conducted by testing in five age groups if the variance of BP over time is statistically significant. A *p* value of <0.05 was considered statistically significant. Python (version 3.2.5) was used for analysis, visualization, and statistical tests. Mann–Whitney *U* test and χ^2^ test were conducted using *scipy.stats* package, Mann–Kendall test was conducted using *PyMannKendall*.

#### Model sensitivity analysis

For task 1, we conducted model sensitivity analyses using an earlier dataset (January 1, 2011, to December 31, 2014) to see if our findings are robust.

### Ethical approval

This study was approved by the Ethical Review Board of Guangdong Second Provincial General Hospital.

## Results

From January 1, 2015, to December 31, 2018, we observed 71,468 eligible health check-up records. Of those, 12.05% (8615/71468) were with high SBP and 6.78% (4849/71468) were with high DBP. Characteristics of the study samples are shown in Table 1. All variables demonstrate significant differences between the high blood pressure group and the normal group. The prevalence of high SBP/DPB in different age groups for men and women is shown in Tables 2, 3.

**TABLE 1:** Characteristics of research samples.

	High blood pressure (*n* = 9032)	Normal blood pressure (*n* = 62436)	Significance test
		
	Mean (SD)/n (%)	Mean (SD)/n (%)	
Age	54.1 (16.5)	37.5 (12.8)	Statistic = 4.4 × 10^8^, *p* = 0.000
BMI (kg/m^2^)	25.3 (3.59)	22.9 (3.34)	Statistic = 3.8 × 10^8^, *p* = 0.000
GLU (mmol/L)	5.60 (1.76)	4.95 (0.96)	Statistic = 3.7 × 10^8^, *p* = 0.000
TG (mmol/L)	1.95 (1.60)	1.44 (1.13)	Statistic = 3.7 × 10^8^, *p* = 0.000
Men	6494 (71.9%)	37524 (60.1%)	χ^2^ = 5.7, *p* = 0.001

**TABLE 2:** Prevalence of high SBP/DBP in different age groups among men.

Age groups	No. of records	No. of high SBP (%)	No. of high DBP (%)
18–39	26934	1766 (6.56%)	1182 (4.39%)
40–49	7026	944 (13.44%)	971 (13.82%)
50–59	4858	1129 (23.24%)	922 (18.98%)
60–69	3083	1139 (36.94%)	584 (18.94%)
70–79	1430	754 (52.73%)	199 (13.92%)
>=80	679	388 (57.14%)	51 (7.51%)

**TABLE 3:** Prevalence of high SBP/DBP in different age groups among women.

Age groups	No. of records	No. of high SBP (%)	No. of high DBP (%)
18–39	16505	150 (0.91%)	201 (1.22%)
40–49	4508	282 (6.26%)	194 (4.30%)
50–59	2997	548 (18.28%)	281 (9.38%)
60–69	2159	792 (36.68%)	195 (9.03%)
70–79	1068	593 (55.52%)	61 (5.71%)
>=80	221	130 (58.82%)	8 (3.62%)

**FIGURE 2:**
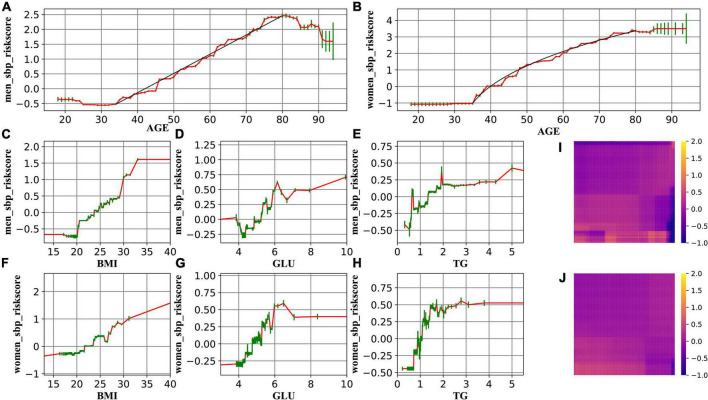
The change in the risk of high SBP related to age (**A** for men and **B** for women), BMI (**C** for men and **F** for women), GLU (**D** for men and **G** for women), and TG (**E** for men and **H** for women). **(I,J)** Demonstrate that the interaction effect is marginal. Red lines represent the spline-based GAM curves. Green lines represent the confidence intervals. Black lines in **A** and **B** are drawn to guide the eyes.

Figures 2A,B demonstrates the relationship between age and the risk of high SBP for men and women, respectively. In general, the risk of high SBP increases with age, reaching a plateau in late life. For high DBP risk, an inverse U-shaped age-related risk trend was identified. Women compared with men exhibited a first-steeper-then-slower increase pattern in BP that began at age 42 years. The ROCAUC was calculated as 0.837 for the main GA^2^M model and 0.803 for the standard logistic regression model. Results of the model validation analysis can be found in Supplementary Figures 1, 2. They exhibit very similar patterns to the main results, indicating that the patterns discovered in this study are robust and generalizable.

**FIGURE 3:**
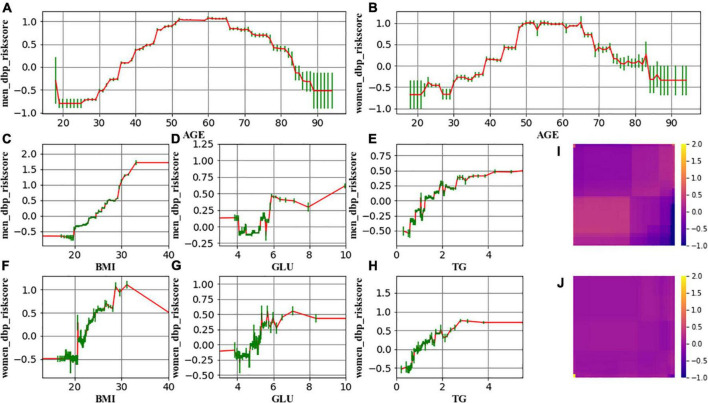
The change in the risk of high DBP related to age (**A** for men and **B** for women), BMI (**C** for men and **F** for women), GLU (**D** for men and **G** for women), and TG (**E** for men and **H** for women). **(I,J)** Demonstrate that the interaction effect is marginal. Red lines represent the spline-based GAM curves. Green lines represent the confidence intervals.

The resulting curves (Figures 2, 3) also enable us to explore the transition points of the role of Age, BMI, GLU, and TG, by identifying the curve where the risk score (*y*-axis) rises above 0. Results of the transition point are reported in Tables 4, 5.

**TABLE 4:** Transition points for age, BMI, GLU, and TG in terms of the risk of high SBP.

	Men	Women
Age group	40–50	40–50
BMI interval	20–25	20–25
GLU interval (mmol/L)	4–6	4–6
TG (mmol/L)	1.4	1

**TABLE 5:** Transition points for age, BMI, GLU, and TG in terms of the risk of high DBP.

	Men	Women
Age group	40–50; 80–85	40–50; 80–85
BMI interval	20–25	20–25
GLU interval (mmol/L)	4–6	4–6
TG (mmol/L)	1.3	0.9

**FIGURE 4:**
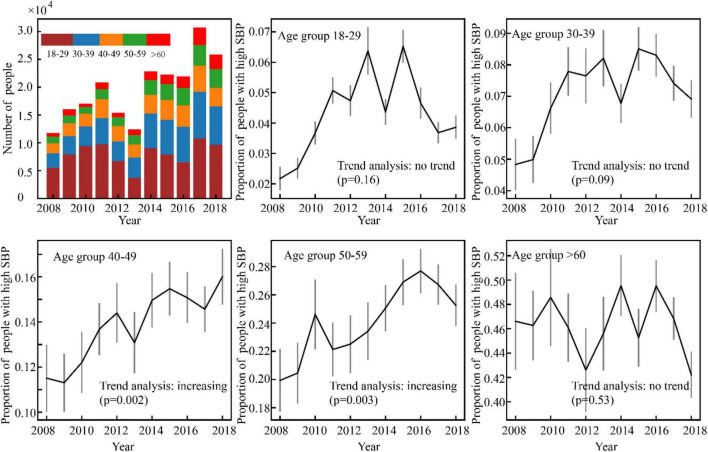
Variance of the number of high SBP men with time.

**FIGURE 5:**
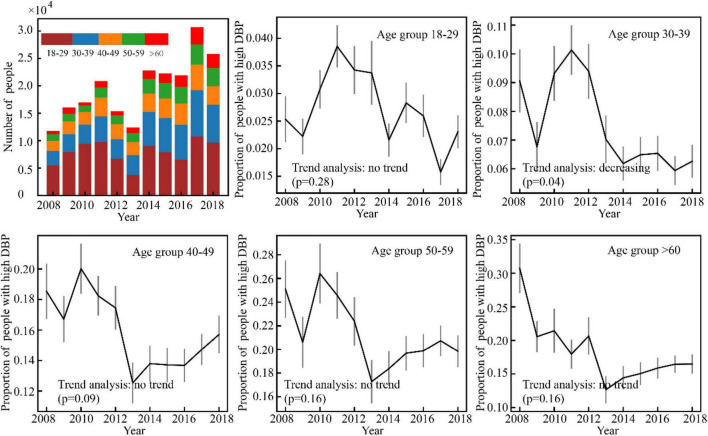
Varience of the number of high DBP male participants with time in Guangdong, China (N = 44010).

**FIGURE 6:**
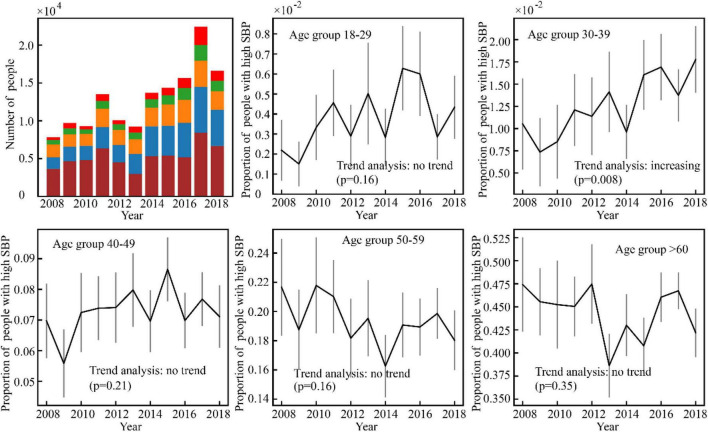
Variance of the number of high SBP women with time.

**FIGURE 7:**
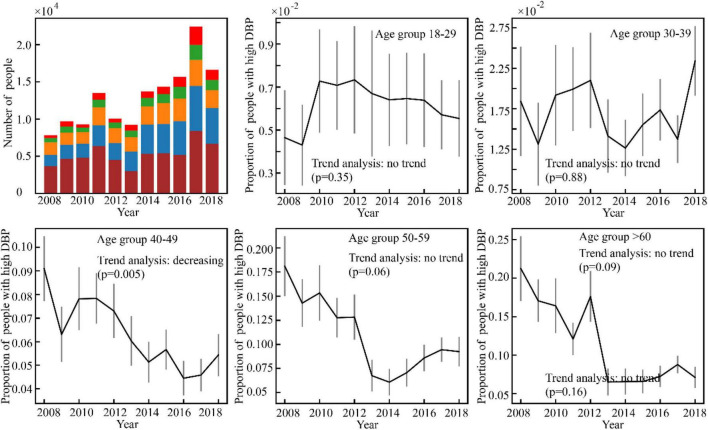
Variance of the number of high DBP women with time.

Results of the high BP prevalence for each age group during the study period (2008–2018) were reported in Figures 4–7. For men, an increasing trend of high SBP risk was observed among those aged 40–49 years (*p*0.002) and 50–59 years (*p*0.002), and high DBP risk decreased among those aged 30–39 years (*p*0.004). For women, an increasing trend of high SBP risk was witnessed among those aged 30–39 years (*p*0.008), and high DBP risk decreased (*p*0.005) among those aged 40–49 years. No significant trend in other age groups was observed.

## Discussion

Our study depicted the age-related trend of risk in high SBP/DBP and identified the specific age group which showed significant changes in risk in high SBP/DBP prevalence over time among male and female adults. The subjects in this study showed a rise in SBP from age 35 to 79 years and a concurrent early increase in DBP; after age 50–65 years, DBP declined. GA^2^M analysis of high SBP/DBP risk as a function of age, adjusting for BMI, GLU, and TG, showed that slope patterns of age-risk curves differed for men and women, whereas it was curvature that differed for SBP and DBP. A general picture of the BP trend in south China was also reported. For men aged 40–59 years and women aged 30–39 years, an increasing trend of high SBP was observed. For men aged 30–39 years and women aged 40–49 years, the trend of high DBP was deflated.

The present study indicated that the risk of SBP rises linearly with age for men, whereas it was a non-linear relationship between age and SBP risk for women. More concretely, SBP risk progresses more rapidly in the age period 35–55 (first phase) than lifetime thereafter (55–80, second phase). This finding differs from previous literature where a linear rise for both genders was reported (5, 7). From a methodological perspective, such discrepancy appears probably because (a) literature of this line manually splits continuous age into discrete age groups, bringing in human influence, and (b) through counting the number of subjects that fall into handcrafted age intervals, there lacks adjustment for confounding factors. This study took a step forward by leveraging the strength of GA^2^M that permits arbitrary complex relationships between dependent and independent variables. The resulting c-statistics of GA^2^M and a standard logistic regression model echoes our hypothesis that GA^2^M enables a more precise way of depicting the feature–target relationship. Plausible explanations from the physiological perspective would be that (a) menopause might be attributable to the disparity between the two phases; (b) taking men as a reference, previous literature reports that women’s blood pressure starts lower than men’s, catches up around age 45, and frequently becomes slightly higher thereafter (5, 10). Therefore, SBP risk elevates more rapidly in the first phase to catch up to BP levels in men.

GA^2^M analysis also enables us to quantify the transition point of the role of covariates. Such information can signal us when we should pay special attention to the measurement. For example, the transition point of the role of BMI in SBP is located in 20–25, indicating a protective role of BMI ≤ 20 in preventing SBP, and a harmful role of BMI ≥ 25 in inducing SBP. This happened to echo the stratification of BMI index alone. It is noteworthy that the transition point (80–85) in DBP seen in the elderly is probably the result rather than the cause of the disease process. Age-related stiffening of the aorta is associated with a decreased capacity of the elastic reservoir and hence a greater peripheral runoff of stroke volume during systole. Thus, with less blood remaining in the aorta at the beginning of diastole, and with diminished elastic recoil, diastolic pressure decreases with increased steepness of diastolic decay (5). The exaggerated fall in DBP risk seen in the elderly suggests a process of transmural pressure-induced arterial wall damage resulting in large artery stiffness, enforcing age ≥85 to be a phantom “protective role.” Apart from this observation, there is a marginal difference for SBD and DBP, and for men and women with respect to the role of covariates in high BP risk. Generalized additive models have recently been used to explore the association between physical activity and mental health burden (14), to investigate the association of depression with dietary inflammatory index (15), and to predict postoperative acute kidney injury and acute respiratory failure (16). This study adds the age–blood pressure relationship to a growing list of fields of research that are actively capitalizing on GAM.

With respect to the second goal of this article, we observed an increasing trend of high SBP risk among middle-aged people during the very recent decade. This finding echoes previous evidence that the incidence of hypertension has been shifting toward younger generations (11, 12). Results also indicate that there exists gender discrepancy in terms of those experiencing an increasing high SBP risk: Age groups of men are older and wider compared to women (40–59 vs. 30–39). Plausible factors leading to such discrepancy would be sex hormones, lifestyles (e.g., alcohol intake) (17–19), social pressures (12), etc.

This study has implications for hypertension management and prevention from both disease and population perspectives. From the disease perspective, our results support the evidence that aging is an important risk factor for hypertension, for constant increase of risk in high SBP with age. According to the trends of risk in high SBP and high DBP, diagnosis of hypertension could become more dependent on SBP measurement among the elderly. From the population perspective, our study supports the threshold for significant changes in blood pressure at 40–45 years of age. The current national guideline for hypertension management in China (2019) defined people aged over 45 years as a high-risk groups for hypertensions. However, no recommendations for regular blood pressure measurement and hypertension screening have been provided for Chinese adults. Another implication informs the importance of controlling elevated blood pressure among middle-aged Chinese adults (30–59 years old) who might be the main contributors of trends of people suffering from hypertension at a younger age. About 30% of middle-aged people in China were estimated to suffer from hypertension (20), while 87% of them with ISH remain untreated (21). Future research on improving awareness, prevention, and treatment of hypertension among middle-aged adults in China could be taken.

Although analyses of this study were based on a well-constructed model and a large volume dataset, several inherent deficits of routine health check-up data should be noted when interpreting the results. First, a check-up BP record relies on a one-off BP test, but tests conducted more than one time on different days are recommended to acquire more solid BP results. Secondly, this study reports findings from a single center, whether it is generalizable to populations elsewhere needs further investigation.

## Conclusion

In contrast with the notion that systolic blood pressure linearly increases with age in individuals, our results suggested that SBP risk progresses more rapidly in early lifetime in women, compared to lifetime thereafter. Thresholds of the increasing trend of SBP suggest a possible need for hypertension screening in China after the age of 40. The changing risk of high BP in different age groups in the past decade (from 2008 to 2018) suggested the necessity of controlling elevated blood pressure of middle-aged Chinese adults to avoid the rejuvenation of hypertension among younger adults.

## Data availability statement

The original contributions presented in this study are included in the article/Supplementary material, further inquiries can be directed to the corresponding authors.

## Ethics statement

The studies involving human participants were reviewed and approved by the Ethical Review Board of Guangdong Second Provincial General Hospital. Written informed consent for participation was not required for this study in accordance with the national legislation and the institutional requirements.

## Author contributions

WC, ZX, and JT: conceptualization. YD, WC, and ZX: writing – original draft. ZX, YD, WC, QZ, XW, CH, JH, FJ, MG, and HR: review and editing. WC, QZ, and JT: supervision. WC and JT: project administration and funding acquisition. All authors contributed to the article and approved the submitted version.
