# Supplementary material for: Age-related changes in the risk of high blood pressure
Source: Front Cardiovasc Med. 2022 Sep 15;9:939103. doi: 10.3389/fcvm.2022.939103 (PMC9521719; doi:10.3389/fcvm.2022.939103)
Supplement: Supplementary file 1 [file Data_Sheet_1.docx]

**Appendix:**

**Results of the** **sensitivity analysis**

During the model sensitivity analysis period of Jan 1, 2015-Dec 31, 2018, 10552 eligible records were obtained. Of those, 16.06% (1695/10552) are with high SBP and 15.31% (1616/10552) are with high DBP. Results of the model validation analysis didn’t change the general picture of the main results.

**
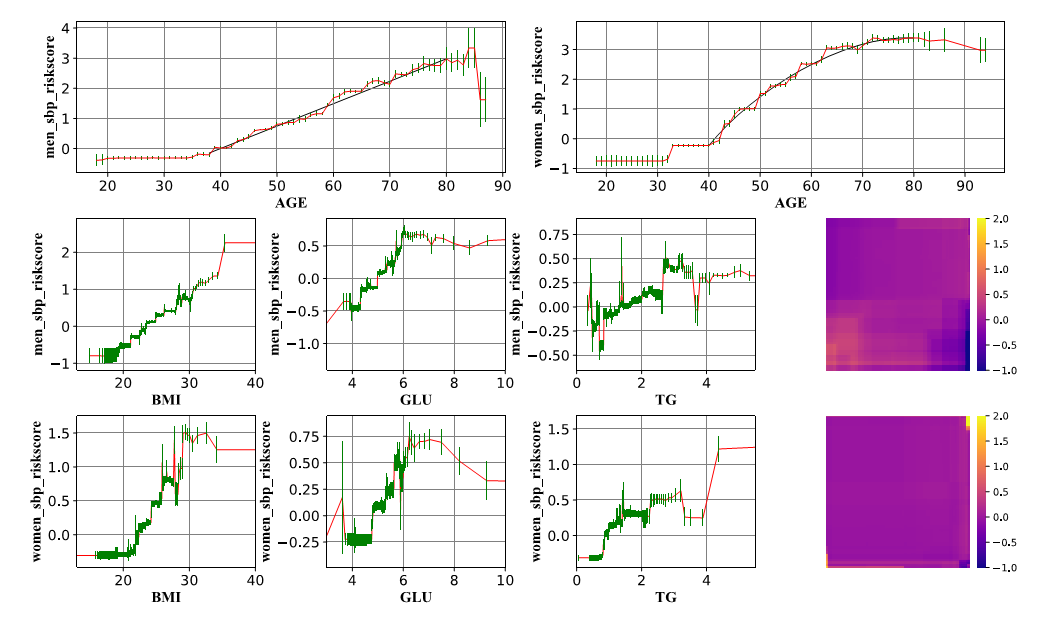
**

**Supplementary Figure 1. The change of the risk in High SBP related to age and other covariates (Model validation). Black lines were drawn to guide the eyes.**

**
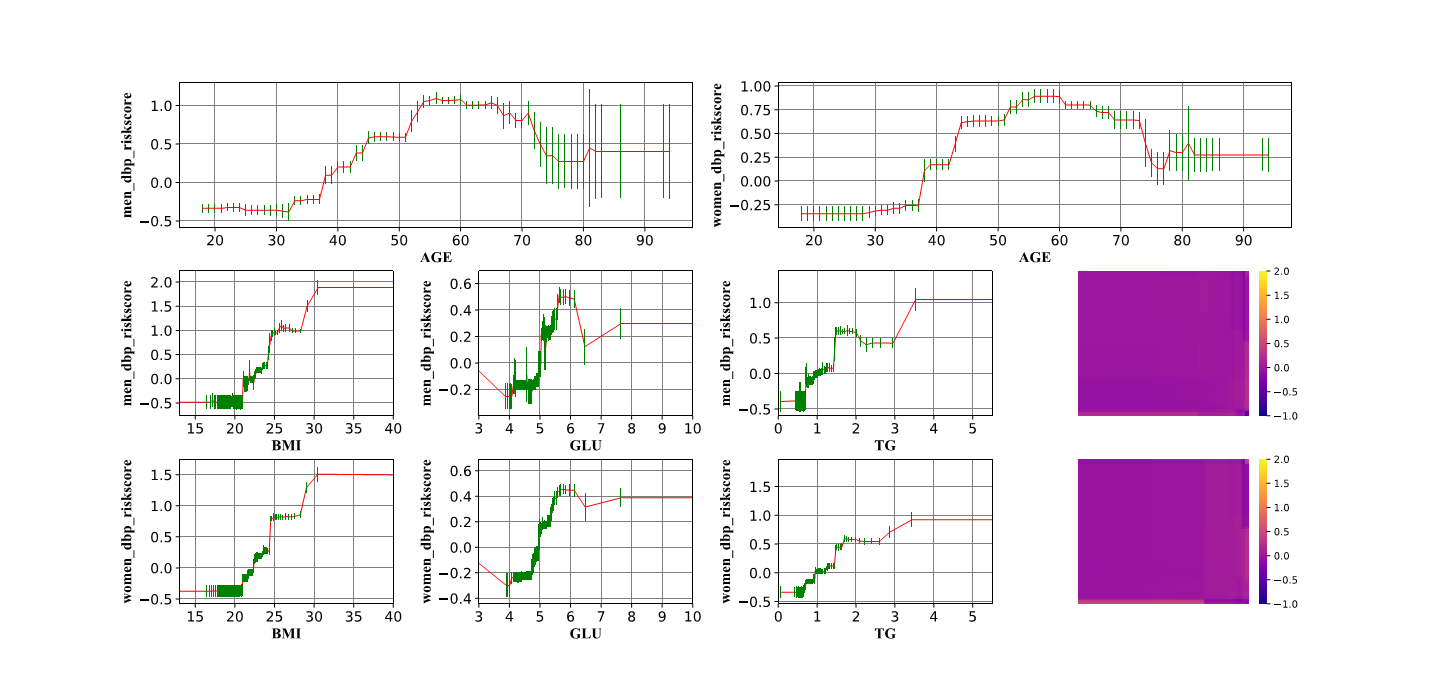
**

**Supplementary Figure 2. The change of the risk in High DBP related to age and other covariates. (Model validation)**
